# miR-1338-5p Modulates Growth Hormone Secretion and Glucose Utilization by Regulating *ghitm* in Genetically Improved Farmed Tilapia (GIFT, *Oreochromis niloticus*)

**DOI:** 10.3389/fphys.2017.00998

**Published:** 2017-12-06

**Authors:** Jun Qiang, Jing Wen Bao, Hong Xia Li, De Ju Chen, Jie He, Yi Fan Tao, Pao Xu

**Affiliations:** ^1^Key Laboratory of Freshwater Fisheries and Germplasm Resources Utilization, Ministry of Agriculture, Freshwater Fisheries Research Center, Chinese Academy of Fishery Sciences, Wuxi, China; ^2^Wuxi Fisheries College, Nanjing Agricultural University, Wuxi, China

**Keywords:** microRNA, tilapia, functional analysis, growth hormone, insulin, loss of growth

## Abstract

MicroRNAs (miRNAs) are endogenous, non-coding small RNA molecules about 22 nt in length, which could regulate the expressions of target genes and participate in growth and development of organisms. Genetically improved farmed tilapia (GIFT, *Oreochromis niloticus*) is an important economic freshwater species in China and the growth performance is one of the main breeding indicators. *Growth hormone inducible transmembrane protein* (*ghitm*) plays an important role in growth and development of both mammals and invertebrates; however, little studies have been reported on fish. Our previous experiments indicated that miR-1338-5p expression may be negatively correlated with *ghitm* expression. In this study, we firstly used qRT-PCR and northern blot to verify the expression of miR-1338-5p and *ghitm*, and determined the binding site of miR-1338-5p in the *ghitm* 3′-untranslated region (UTR) by luciferase reporter assay. Secondly, juveniles GIFT injected with miR-1338-5p antagomir were used to analyze the regulatory function of the miR-1338-5p-*ghitm* pair *in vivo*. The results showed that the *ghitm* 3′-UTR was complementary to the 5′ 2–8-nt site of miR-1338-5p. Inhibition of miR-1338-5p promoted *ghitm* expression in the pituitary and liver of GIFT. *ghitm* could interfere in the growth hormone (Gh)–growth hormone receptor (Ghr)–insulin-like growth factor (Igf) signaling pathway by competing with the *ghr1* for combination with Gh, and then reduce the growth of GIFT. Moreover, the reduction of Gh in serum may regulate insulin secretion and result in the increasing sugar and fat storage in serum and liver. Our results suggest that miR-1338-5p participates in the growth and development of GIFT through the regulation of *ghitm*, which provides theoretical support for the study of the fish growth mechanism.

## Introduction

Growth hormone (Gh) can not only enhance the appetite, promote feed utilization, and accelerate growth of fish, but also plays an important role in regulating the energy metabolism, osmotic pressure, reproduction, behavior, and immunoregulation of fish (Sakamoto et al., 1997; Johansson et al., 2004; Møller et al., 2007; Rousseau and Dufour, 2007; Benedet et al., 2010). Therefore, Gh is one of the most potent and effective growth promoters in fish culture (Yowe and Epping, 1995). Gh can promote growth in two ways. One is through the *gh*–*gh receptors* (*ghrs*)–*insulin-like growth factor* (*igf*) axis, which is a regulatory pathway that has been widely confirmed in vertebrates (Butler and LeRoith, 2001). The other way is the direct activation of the target cell membrane receptor or transmembrane protein by Gh to achieve its biological effects (Li et al., 2001; Yoshida et al., 2006; Gao et al., 2014). The direct Gh regulation has been shown to mediate growth, development, and tumor formation in laboratory animals, but the specific biological function and mechanism are not yet clear.

*Growth hormone inducible transmembrane protein* (*ghitm*) was first discovered in transgenic mice treated by Gh antagonists (Li et al., 2001). *ghitm* was present mainly in adipose tissue of the test mice. The expression level of *ghitm* in the Gh antagonist-treated mice was significantly higher than in the control mice, and Gh could induce and regulate the activity of Ghitm. *ghitm* was expressed in mouse embryos and differentiated tissues, as well as in mammalian cell lines (Yoshida et al., 2006). The body weight of Gh antagonist-treated mice was significantly higher than that of control mice with the same food intake, and was mainly due to an increase in body fat, suggesting that Ghitm may be involved in regulating animal growth and development (Knapp et al., 1994). In addition, Ghitm was associated with the growth and age of *Drosophila melanogaster* and played a role in the anti-aging mechanism of oxygen free radicals (Zou et al., 2000). There are relatively few studies of *ghitm* in aquatic animals, only purple sea urchin (*Anthocidaris crassispina*) (Hibino et al., 2006), pacific oyster (*Crassostrea gigas*) (Zhang et al., 2012), pearl oyster (*Pinctada martensii*) (Luo et al., 2016), and sea cucumber (*Apostichopus japonicus*) (Gao et al., 2014) have been studied so far. For instance, *ghitm* was most highly expressed in the gonad of pearl oyster, followed by the hepatopancreas and adductor muscle, all of which likely participate in the growth and development of pearl oysters (Luo et al., 2016). It is unclear whether *ghitm* has a similar function in fish. Exploring the response and regulatory mechanisms of *ghitm* will help in better understanding fish growth and physiological characteristics.

MicroRNAs (miRNAs) are a class of non-coding small RNAs that are about 22 nt in length. They are involved in the growth and development of organisms by post-transcriptional regulation of gene expression (Bartel, 2004; Chen et al., 2010). With the development of high-throughput sequencing technology, the identification and functional studies of miRNAs in fish have advanced. For example, 140 conserved and 66 differentially expressed miRNAs in the early development of metamorphosis in Japanese halibut (*Paralichthys olivaceus*) (Fu et al., 2011). miR-1, miR-21, miR-26a, miR-27a4, and miR-222 were differentially expressed in skeletal muscle at different developmental stages in carp (*Cyprinus carpio*) (Yan et al., 2012). These differentially expressed miRNAs may be related to muscle development. Three-hundred and forty seven conserved and twenty-seven differentially expressed miRNAs involved in growth of blunt snout bream (*Megalobrama amblycephala*) (Yi et al., 2013). In addition, between fast-growing and slow-growing groups of mandarin fish (*Siniperca chuatsi*), miR-122, miR-192, miR-451, and let-7j-5p may be involved in growth, development, metabolism, and signal transduction (Wang et al., 2017).

Growth performance is one of the most important indicators in the tilapia breeding process. Genetically improved farmed tilapia (GIFT, *Oreochromis niloticus*) is the most widely cultivated tilapia in China, and its growth rate is 40% faster than that of other strains of Nile tilapia, and 25% faster than that of hybrid tilapia (*O. niloticus* × *Oreochromis aureus*) (Qiang et al., 2014, 2015). Therefore, GIFT is an important model organism to study the growth regulation mechanism. In our previous study, we initially screened miRNAs associated with development in GIFT under heat stress, including let-7j, let-7d-5p, miR-99, miR-16b-3p, miR-1338-5p, and miR-730a-5p (Qiang et al., 2017a). The function of miR-1338-5p had not been reported in any species. We used the miRanda v3.3a toolbox (http://www.microrna.org/microrna/home.do) to predict the possible target genes of miR-1338-5p. We found that the 3′-untranslated region (UTR) of *ghitm* was completely complementary with miR-1338-5p, and we also confirmed this negative complementary pairing by transcriptome sequencing. The regulatory mechanism of the miR-1338-5p-*ghitm* pair in the growth and development of tilapia and how it is controlled need further exploration.

To investigate this problem, we assumed that the miR-1338-5p-*ghitm* pair may mediate signal transduction in the growth and development of GIFT through a series of biological pathways. To verify this hypothesis, we first validated the pairing relationship by qRT-PCR, northern blot, and luciferase reporter assay. Then, we studied the regulation of *ghitm* and changes of downstream regulators by inhibition of miR-1338-5p. This study will provide technical guidance for the breeding of fast-growing GIFT, and a scientific basis for the growth of other fish species.

## Materials and methods

In this study, all experimental procedures involving fish were performed according to the protocol approved by the Freshwater Fisheries Research Centre (FFRC) of the Chinese Academy of Fishery Sciences, Wuxi, China.

### Experimental fish

GIFT were selected from the Yixing tilapia base of FFRC (Wuxi, China). We chose fish that were strong and disease- and injury-free, as the experimental fish. Before the experiment, the fish were held in an indoor water recycling system (water temperature 27°C ± 0.3) with continuous aeration, and natural photoperiod for 10 days. Submerged feed (crude protein 35.0%, fat 8.0%) at 8% of body weight was fed three times a day (7:00, 11:00, and 16:00). Dissolved oxygen was higher than 5 mg L^−1^, pH was 7.4 ± 0.2, and ammonia nitrogen and nitrite levels were lower than 0.1 mg L^−1^ during the experiment.

### Experimental design

#### Verification of relationship between miR-1338-5p and its potential target gene

We selected samples (including control group and 24 h heat-stressed group) that were stored from our previous experiment, and extracted RNA (Qiang et al., 2017a). The expression patterns of miR-1338-5p and its potential target gene *ghitm* were analyzed by qRT-PCR. A northern blot experiment was conducted as described by Zhang et al. (2008). Briefly, total RNA was isolated using a 15% PAGE, 8 M urea denatured gel. The membrane was transferred at 0.6 mA/cm^2^ with constant flow for 4 h to a N^+^Nylon membrane, and the miR-1338-5p probe was labeled with γ-^32^P at 37°C for overnight hybridization. The miR-1338-5p probe sequence was 5′-CATTCTCAGGTTGGACAGTCCT-3′. The U6 probe, which was used as the internal control, was provided by Signosis, Inc. (Sunnyvale, USA). The miR-1338-5p probe was synthesized by Shanghai Biotech Bioengineering Technology Service Co., Ltd (Shanghai, China). The MiRNA Northern Blot kit was purchased from Signosis, Inc.

#### Determination of miR-1338-5p expression in six GIFT tissues

Eight healthy GIFT (28.73 ± 1.02 g) were treated with 200 mg L^−1^ MS-222 for rapid deep anesthesia. Blood, liver, spleen, intestine, pituitary, and muscle were dissected out, cooled in liquid nitrogen, and stored in a–80°C refrigerator for further miR-1338-5p tissue expression studies.

#### Verification of the miR-1338-5p binding site in ghitm by luciferase reporter assay

The *ghitm* 3′-UTR sequence was synthesized and inserted into the downstream region of the firefly luciferase gene in a pGL3-control vector (Promega, Madison, USA). We constructed a reporter gene plasmid (*ghitm*-Wild Type, WT) containing the desired sequence. Seven bases (ACAGTCC) in the seed region of the *ghitm* 3′-UTR were mutated to construct a *ghitm* 3′-UTR mutant (*ghitm*-Mut) (TGCCCAT).

HEK293T (human embryonic kidney) cells were treated by trypsin digestion, and counted. Then, 2.0 × 10^5^ HEK293T cells were inoculated in 24-well plates at 24 h before transfection and incubated in a 37°C, 5% CO_2_ incubator. Each well contained 0.2 μg *ghitm*-WT or *ghitm*-Mut, 0.4 μL X-tremeGENE HP (Roche, Basel, Switzerland), and 0.45 μg miRNA mimics or miRNA negative control (NC). The plasmid was diluted with 40 μL of Opti-MEM medium, then 0.9 μL of X-tremeGENE HP was added, mixed, and allowed to stand at room temperature for 15 min. Finally, 10 μL of the mixture was added gently to each well, and the plates were incubated in a 37°C, 5% CO_2_ incubator. Each treatment has five biological samples, and each sample has duplicate wells.

After transfection for 48 h, the medium was absorbed and 80 μL of diluted 1X cell lysate was added to each well. The plates were placed on a decolorization shaker for 1 h. Then, the cell lysates were collected from each well, and centrifuged at 12,000 g for 1 min to precipitate impurities. After centrifugation, the cell lysates were placed in an opaque 96-well plate, and firefly and Renilla luciferase were added in turn according to the manufacturer's instructions (Promega, Madison, USA), and tested using a BioTek Eon microplate reader at 465 nm (Vermont, USA).

#### Functional analysis of miR-1338-5p in regulating ghitm *in vivo*

A total of 180 juvenile GIFT with average size 5.4 ± 0.4 g were placed randomly in nine 600-L tanks each containing 20 fish. Rearing management was as described in section Experimental Fish. A chemically modified oligonucleotide (miR-1338-5p antagomir: 5′-CATTCTCAGGTTGGACAGTCCT-3′) was synthesized by Guangzhou Ruibo Biological Biotechnology Co., Ltd. (Guangzhou, China). The miR-1338-5p antagomir solution and dilution methods were according to Qiang et al. (2017a). The fish were treated by tail-vein injection of miR-1338-5p antagomir, negative antagomir (seven base mutations in the seed region), or the same volume of 0.2 mol L^−1^ (pH 7.4) phosphate-buffered saline (PBS) at a dose of 50 mg kg^−1^ body mass. Each treatment had three replicates. The fish injected with PBS were the control group. Four fish were selected randomly from each tank at 0 (pre-treatment), 12, 24, and 36 h. The fish were treated with 200 mg L^−1^ MS-222. The pituitary and liver tissues were removed, quickly cooled in liquid nitrogen, and stored in a −80°C refrigerator to analyze miR-1338-5p regulation of *ghitm*.

A total of 270 juvenile GIFT with average size 5.8 ± 0.5 g were placed randomly in nine 600-L tanks each containing 30 fish. Rearing management was as described in section Experimental Fish. Injections of miR-1338-5p antagomir, negative control, or PBS were as described in the previous paragraph. Because the effect time of miRNA antagomir was 7–10 days, to ensure the inhibitory effect, we injected the antagomir once every 6 days. Four fish were selected randomly from each tank at 0 (pre-treatment), 10, 20, and 40 days. After quickly weighing them, a 1.0-ml syringe was used to collect blood from the tail vein. The blood was left to stand in a refrigerator at 4°C for 2 h, then centrifuged at 3,000 × g at 4°C for 10 min. Serum was collected and stored in a −80°C refrigerator for serum biochemical analysis. Additionally, the pituitary and liver tissues were removed, rapidly cooled in liquid nitrogen, and stored in a −80°C refrigerator to study the miR-1338-5p regulatory response mechanism.

### Biochemical analysis of serum and liver

The levels of serum glucose, triglyceride (TG), and total cholesterol (TC) were measured using an BS-400 automatic biochemical analyzer (Mindray, Shenzhen, China). Serum insulin levels were measured as described by Qiang et al. (2017b). Hepatic TG, TC, and glycogen were determined by enzyme-linked immunosorbent assay using test kits from Shanghai Lengton Bioscience. Serum Gh levels were measured using the radioimmunoassay method according to Ayson et al. (1993).

### Expression of miR-1338-5p and *ghitm, gh, ghr1*, and *igf-I* mRNAs by qRT-PCR

Total RNA was extracted from the liver and pituitary tissue samples using Trizol reagent (Invitrogen, CA, USA). A Mir-X™ miRNA First-Strand Synthesis kit and a SYBR® PrimeScript™ miRNA RT-PCR kit (Takara, Dalian, China) were used for the RT reaction and qRT-PCR of miR-1338-5p. The expression of U6 was taken as a reference. Dissociation curve analysis of amplified products was performed at the end of each PCR reaction to confirm that only one PCR product was amplified and detected. For each cDNA, three-well replicates were used. The miRNA specific primer (miR-1338-5p: 5′-AGGACTGTCCAACCTGAGAATG-3′) was synthesized by Genewiz, Inc. (Genewiz, Suzhou, China). The RT reaction and qRT-PCRs of the mRNAs were measured using PrimeScript™ RT Master Mixand SYBR® Premix Ex Taq kits (Takara, Dalian, China). 18S rRNA was used as a reference. The *ghitm, gh, ghr1*, and *igf-I* mRNA primers were synthesized by Shanghai GeneCore Bio Technologies Co., Ltd. (Shanghai, China) and are shown in Table 1. The expression levels of miR-1338-5p and the mRNAs were quantified using an ABI 7900HT Fast Real-Time PCR System (Applied Biosystems, NY, USA), calculated using the 2^−ΔΔCt^ method, and analyzed with Relative Quantification manager software.

**Table 1:** Primer sequences.

**Target mRNA**	**Sequence**	**NCBI Genbank accession no**
*ghitm*	F: 5′-GTGGGAGGTCTGTCTACTGTTGC-3′	XM003441789.3
	R: 5′-TCCGAATGCTGAGGTGGG-3′	
*gh*	F: 5′-TCGGTTGTGTGTTTGGGCGTCTC-3′	XM003442542
	R: 5′-GTGCAGGTGCGTGACTCTGTTGA-3′	
*ghr1*	F: 5′- CATGGCTCTCTCGCCCTCCTCTAA-3′	XM003446082
	R: 5′-ATGTCGTGTGGTCCCAGTCAGTGA-3′	
*igf-I*	F: 5′- TTGTCTGTGGAGAGCGAGGCTT-3′	XM003448059
	F: 5′- CAGCTTTGGAAGCAGCACTCGT-3′	
18S rRNA	F: 5′-GGCCGTTCTTAGTTGGTGGA-3′	JF698683.1
	F: 5′-TTGCTCAATCTCGTGTGGCT-3′	

### Statistical analysis

In this study, values are expressed as mean ± standard error of mean (SEM). The data were analyzed by one-way analysis of variance (ANOVA) using the SPSS 17.0 software. *P-*values < 0.05 were considered to indicate statistically significant differences. Significant differences in different treatments at each sampling point were calculated by Duncan's multiple range tests. Significant differences between values of each treatment obtained at the different sampling times were calculated by paired-sample *t*-tests.

## Results

### Analysis of miR-1338-5p potential target gene and tissue distribution in GIFT

**Figure 1:**
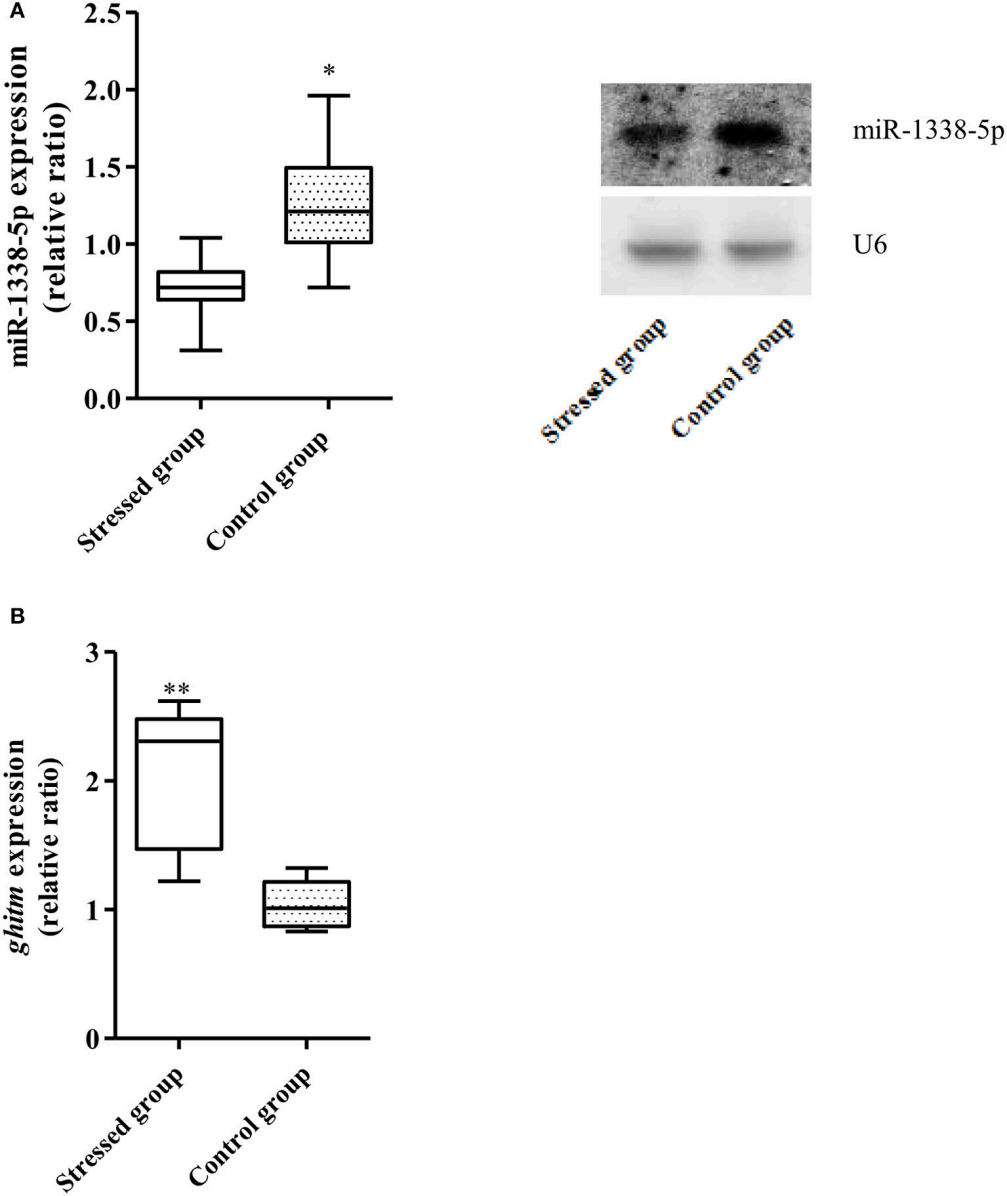
Expression of miR-1338-5p and *ghitm* in GIFT between 37.5°C heat-treated and control samples at 24 h (*n* = 9–12 replicates per group). **(A)** Expression of miR-1338-5p in the heat-stressed and control samples by qRT-PCR and northern blot. Values are expressed as the relative ratio with U6 as an internal control. **(B)** Expression of *ghitm* in the heat-stressed and control samples by qRT-PCR. Values are expressed as the relative ratio with 18S rRNA as an internal control. ^*^P < 0.05 and ^**^P < 0.01, by Duncan's multiple range test.

**Figure 2:**
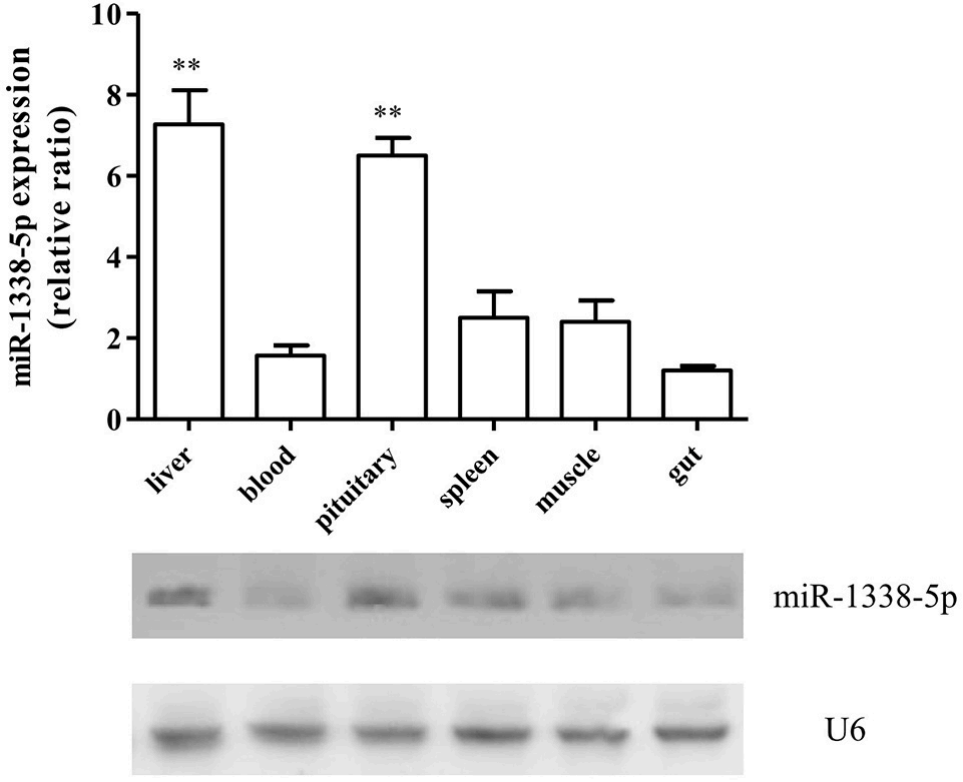
Expression pattern of miR-1338-5p in different GIFT tissues by qRT-PCR and northern blot (*n* = 8 replicates per group). Values are expressed as the relative ratio with U6 as an internal control. ^**^P < 0.01, by Duncan's multiple range test. Fold changes in expression levels were normalized against the levels in gut tissues.

The qRT-PCR results showed that the expression of miR-1338-5p in the heat-treated samples from a previous experiment was significantly decreased and the expression of *ghitm* was significantly increased (Figure 1). Thus, there was a negative relationship between the miR-1338-5p and *ghitm* expression levels in the liver samples of GIFT under heat stress. From the northern blot, we also found that the expression level of miR-1338-5p in the heat-treated group was significantly lower than in the control group (*P* < 0.05). Furthermore, the expression level of miR-1338-5p was significantly higher in liver and pituitary of juvenile GIFT compared with muscle, spleen, blood, and intestinal tissues (*P* < 0.05; Figure 2) by qRT-PCR and northern blot. Therefore, for the functional analysis of miR-1338-5p *in vivo*, we focused mainly on changes of indicators in the liver and pituitary tissues.

### Analysis of the miR-1338-5p binding site in the *ghitm* 3′-UTR

**Figure 3:**
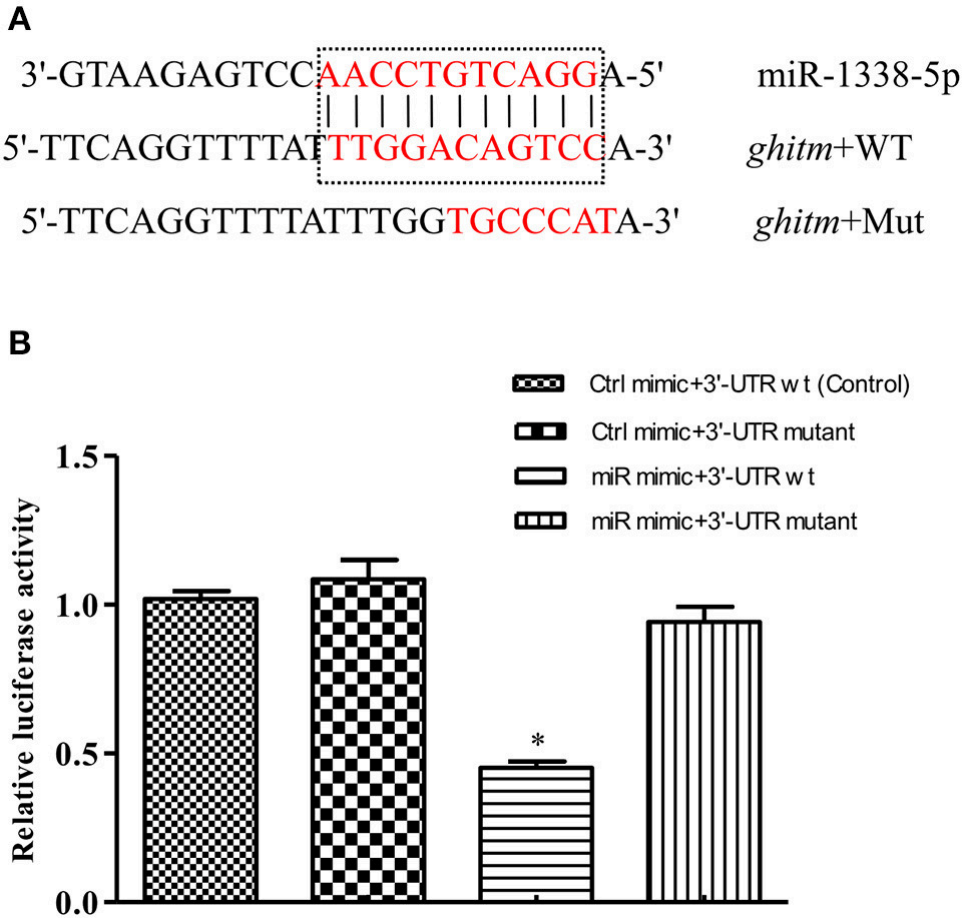
miR-1338-5p regulates *ghitm* expression by binding with the *ghitm* 3′-UTR. **(A)** Sequence complementarity between miR-1338-5p and *ghitm* 3′-UTR, and the mutation site of seed region in miR-1338-5p. **(B)** HEK293T cells and a dual luciferase reporter gene system were applied to verify binding between miR-1338-5p and *ghitm*. Ctrl mimic, 3′-UTR wt, 3′-UTR mutant and miR mimic in the figure are related to the terms miRNA negative control, *ghitm*-WT, *ghitm*-Mut and miR-1338-5p mimic. ^*^P < 0.05, by Duncan's multiple range test.

The complementary binding site between miR-1338-5p and the *ghitm* 3′-UTR was detected by miRanda v3.3a with high score (149) and low free energy (−24.5 kcal/mol) (Figure 3A). A *ghitm* sequence with a seven-base mutation in the 3′-UTR was constructed (Figure 3A). The luciferase activity of *ghitm*-WT+miR-1338-5p mimic was significantly lower than that of *ghitm*-WT+miR-1338-5p NC (P<0.05) (Figure 3B). However, there were no significant differences (P>0.05) in the luciferase activity among *ghitm*-WT+miR-1338-5p NC, *ghitm*-Mut +miR-1338-5p NC, and *ghitm*-Mut+miR-1338-5p mimic, which indicated that the miR-1338-5p mimic bound to the 3′-UTR in the *ghitm*-WT plasmid, and then had an inhibitory effect.

**Figure 4:**
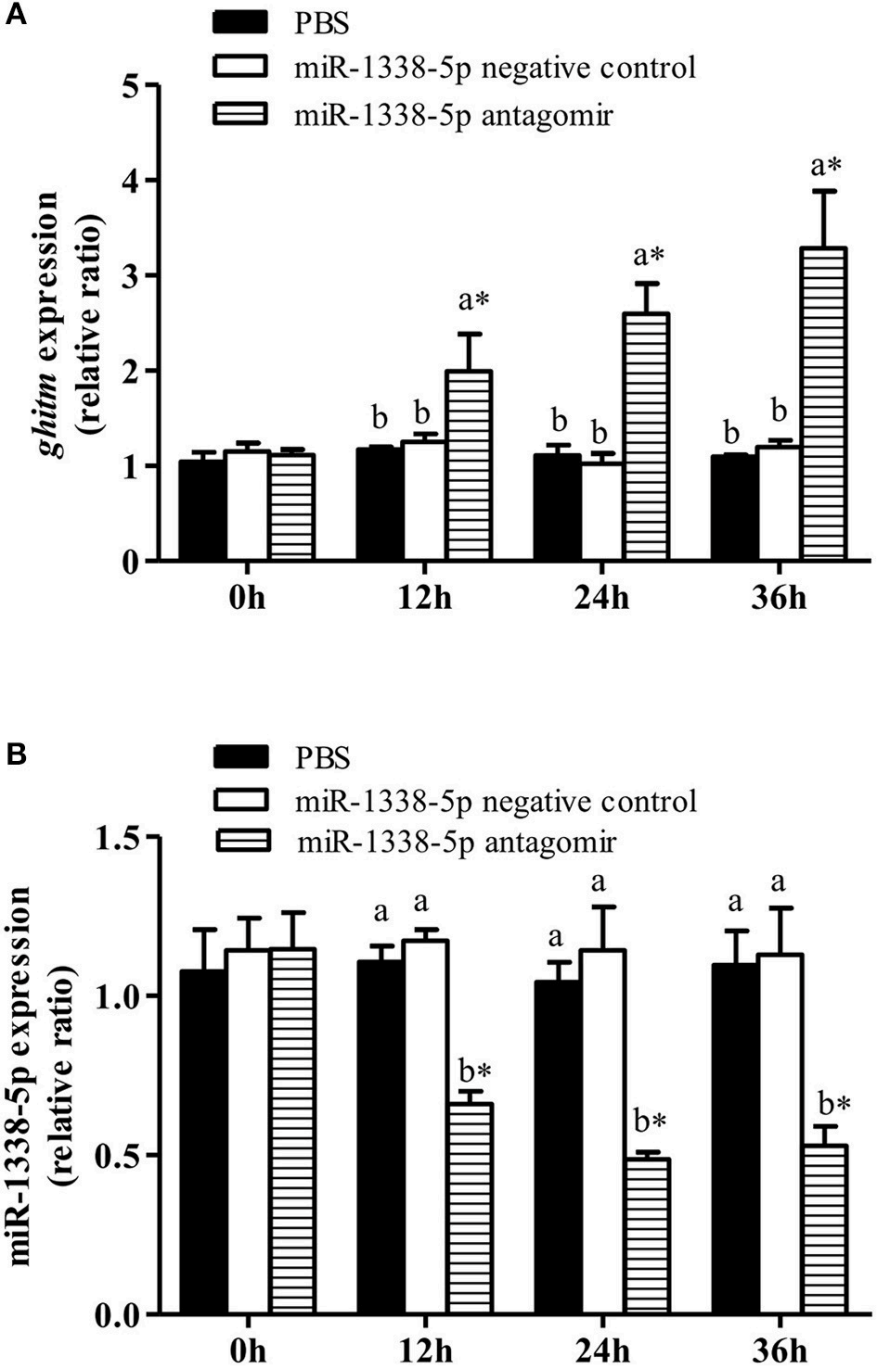
Effect of inhibiting miR-1338-5p on the expression of miR-1338-5p and *ghitm* in GIFT liver over a 36 h period. Juvenile GIFT weighing about 5.4 g received a tail-vein injection of PBS, miR-1338-5p negative control, or miR-1338-5p antagomir at a dose of 50 mg kg^−1^ body weight. qRT-PCR analysis was used to detect the relative expression of *ghitm* **(A)** and miR-1338-5p **(B)** (*n* = 9–12 fish per group). The GIFT injected with PBS were taken as the control. ^*^P < 0.05 indicates significant differences between values of each treatment obtained before and after injection by paired-samples *t-*test. Diverse lowercase letters show significant differences (*P* < 0.05) in different treatments of each sampling point by Duncan's multiple range test. Fold changes in expression levels were normalized against the pre-treatment sampling point of PBS group.

The GIFT were treated with miR-1338-5p antagomir by tail vein injection *in vivo* to analyze the regulatory function of miR-1338-5p (Figure 4). After 12 h, miR-1338-5p expression was significantly lower in the antagomir group compared with the NC and PBS groups, whereas *ghitm* expression was significantly higher in the antagomir group than in the other two groups (*P* < 0.05). This result further indicates a negative regulatory relationship between miR-1338-5p and *ghitm*.

### The miR-1338-5p antagomir regulates miRNA and mRNA expression levels in liver and pituitary of juvenile GIFT

**Figure 5:**
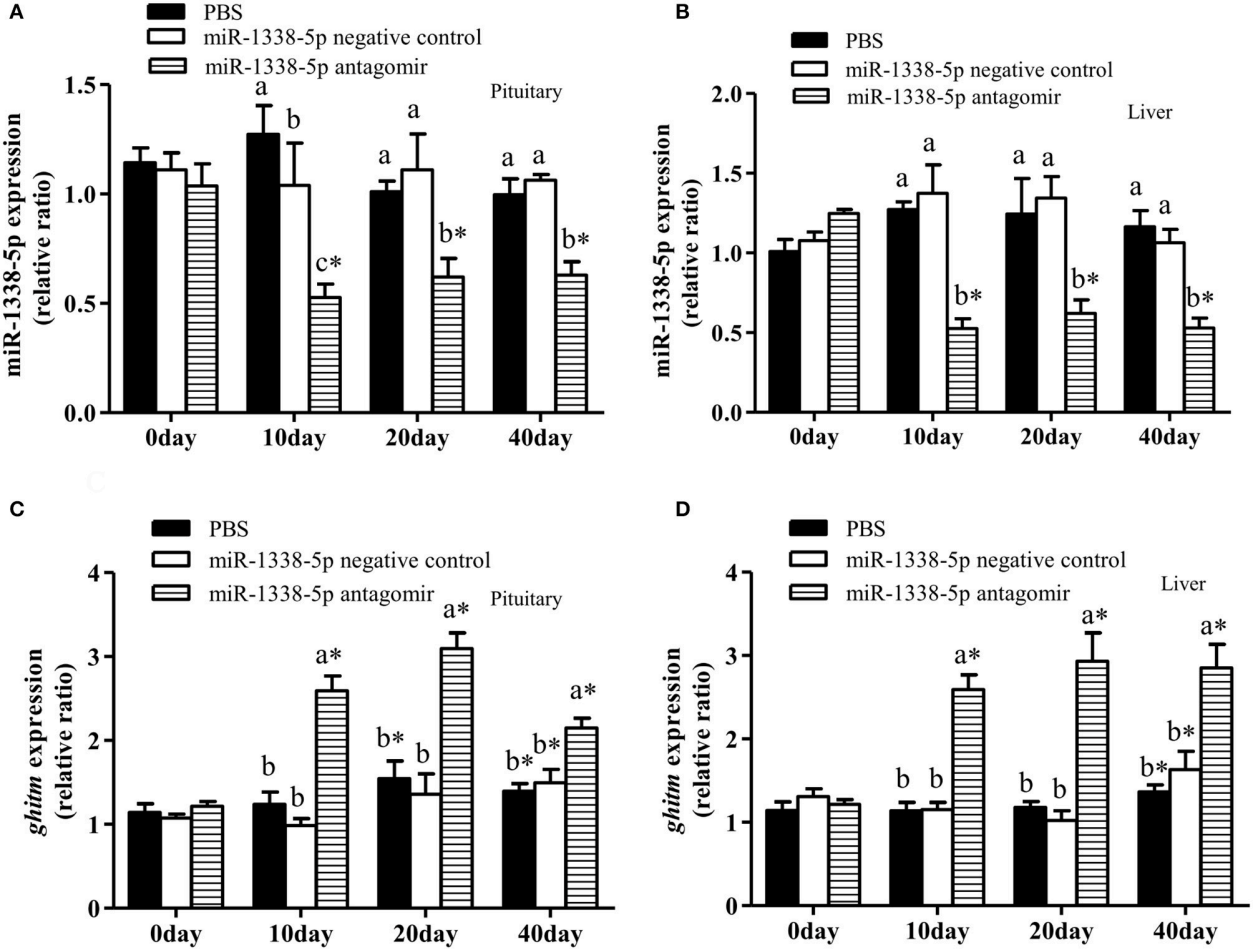
Effect of inhibiting miR-1338-5p on the expression of miR-1338-5p and *ghitm* in pituitary and liver of GIFT over 40 days time period. Juvenile GIFT weighing about 5.8 g received a tail-vein injection of PBS, miR-1338-5p negative control, or miR-1338-5p antagomir at a dose of 50 mg kg^−1^ body weight. The antagomir was injected once every 6 days. qRT-PCR analysis was used to detect the relative expression of miR-1338-5p **(A,B)** and *ghitm* **(C,D)** (*n* = 9–12 fish per group). The GIFT injected with PBS were taken as the control. ^*^P < 0.05 indicates significant differences between values of each treatment obtained before and after injection by paired-samples *t-*test. Diverse lowercase letters show significant differences (*P* < 0.05) in different treatments of each sampling point by Duncan′s multiple range test. Fold changes in expression levels were normalized against the pre-treatment sampling point of PBS group.

The expression levels of miR-1338-5p in liver and pituitary of the antagomir group were significantly lower than those in the NC and PBS groups 10, 20, and 40 days after injection (Figures 5A,B), indicating that the miRNA antagomir inhibited miR-1338-5p expression. The expression levels of *ghitm* in liver and pituitary of the antagomir group were significantly higher than those in the NC and PBS groups at each time point after injection (*P* < 0.05) (Figures 5C,D). However, the expression level of *ghitm* in pituitary of the antagomir group at 40 d was significantly lower than the expression levels at 10 and 20 days, and the expression levels of *GHITM* in pituitary and liver of the PBS and NC groups at 20 and 40 days were significantly higher than those pre-treatment (0 h; *P* < 0.05; Figures 5C,D).

**Figure 6:**
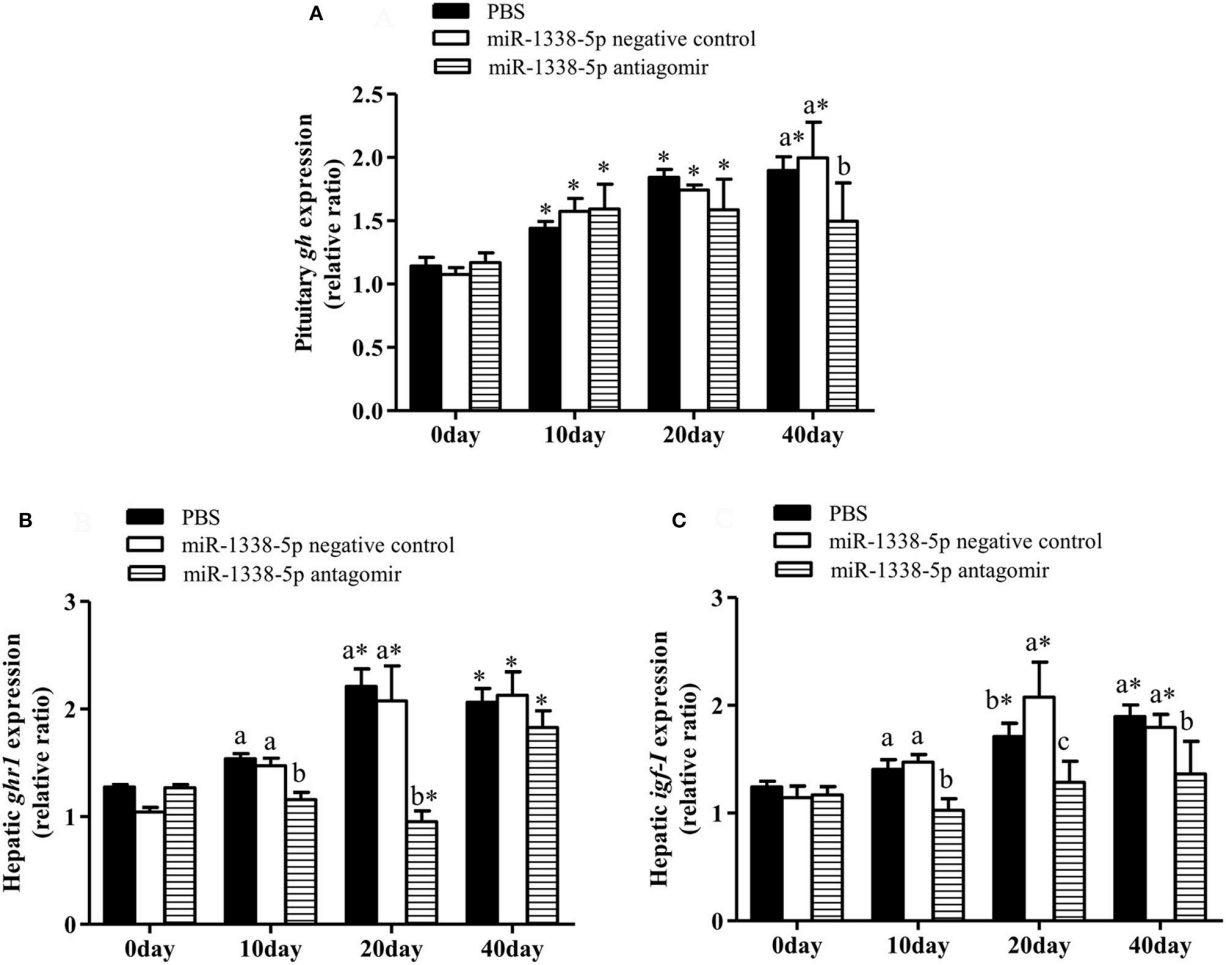
Effect of inhibiting miR-1338-5p on the expression of *gh, ghr*, and *igf-I* in pituitary or liver of GIFT over 40 days time period. Juvenile GIFT weighing about 5.8 g received a tail-vein injection of PBS, miR-1338-5p negative control, or miR-1338-5p antagomir at a dose of 50 mg kg^−1^ body weight. The antagomir was injected once every 6 days. qRT-PCR analysis was used to detect the relative expression of *gh* **(A)**, *ghr1* **(B)**, and *igf-I* **(C)** (*n* = 9–12 fish per group). The GIFTs injected with PBS were taken as the control. ^*^P < 0.05 indicates significant differences between values of each treatment obtained before and after injection by paired-samples *t*-test. Diverse lowercase letters show significant differences (*P* < 0.05) in different treatments of each sampling point by Duncan's multiple range test. Fold changes in expression levels were normalized against the pre-treatment sampling point of PBS group.

At 10 and 20 days after injection, the expression of hepatic *ghr1* in the antagomir group was significantly lower than in the PBS and NC groups (*P* < 0.05) (Figure 6B), and the expression of hepatic *igf-I* in the antagomir group was significantly lower than in the PBS and NC groups at 10, 20, and 40 days (Figure 6C). *gh* expression in the pituitary tissue of the antagomir group was lower than in the PBS and NC groups(*P* < 0.05) at 40 day (Figure 6A). Whereas, there were no significant differences in *gh* expression among the experimental groups at 10 and 20 days (*P* > 0.05). With the prolongation of the rearing time, the expression levels of *gh, ghr1*, and *igf-I* significantly increased in the PBS and NC groups (*P* < 0.05).

### Injection of miR-1338-5p antagomir regulates biochemical indicators of liver and serum in juvenile GIFT

There were no significant differences in body weight of GIFT at 10 and 20 days (Table 2); however, after 40 day of rearing, the body weight of fish in the miR-1338-5p antagomir group was significantly lower than in the PBS and NC groups. Further, at 40 day, the levels of serum TC and TG and hepatic TC in the antagomir group were significantly higher than in the other treatment groups (*P* < 0.05; Tables 2, 3). No differences in the levels of hepatic TG were found among the three treatment groups at 40 day (*P* > 0.05). The levels of hepatic glycogen and serum glucose in the antagomir group at 20 and 40 days were significantly higher than those in the PBS and NC groups, whereas the serum Gh and insulin levels in the antagomir group were significantly lower than those in the PBS or NC groups (*P* < 0.05).

**Table 2:** Growth and biochemistry parameters in juvenile GIFT injected by PBS, miR-1338-5p antagomir, or negative control for 40 days.

	**PBS**	**miR-1338-5p antagomir**	**Negative control**
**GROWTH PARAMETERS (*****n*** = **12 REPLICATES PER GROUP)**			
Fish weight for 10 days (g)	15.17 ± 0.65	16.62 ± 0.87	17.51 ± 0.71
Fish weight for 20 days (g)	23.18 ± 1.62	21.36 ± 1.18	23.80 ± 2.41
Fish weight for 40 days (g)	44.54 ± 2.64^a^	35.13 ± 2.42^b^	47.52 ± 3.21^a^
**LIVER PARAMETERS (*****n*** = **12 REPLICATES PER GROUP)**			
TG for 10 days (mmol·L^−1^)	12.62 ± 0.69	13.41 ± 1.73	13.17 ± 0.95
TG for 20 days (mmol·L^−1^)	14.67 ± 0.73^ab^	13.54 ± 0.72^b^	15.33 ± 1.48^a^
TG for 40 days (mmol·L^−1^)	16.67 ± 1.52	16.94 ± 1.47	17.38 ± 2.13
TC for 10 days (mmol·L^−1^)	2.13 ± 0.19^b^	2.05 ± 0.41^b^	2.52 ± 0.23^a^
TC for 20 days (mmol·L^−1^)	3.67 ± 0.36	3.93 ± 0.79	3.59 ± 0.51
TC for 40 days (mmol·L^−1^)	4.78 ± 0.69^b^	5.91 ± 0.49^a^	5.12 ± 0.63^b^
Glycogen for 10 days (mmol·L^−1^)	2.16 ± 0.17	2.45 ± 0.14	2.22 ± 0.19
Glycogen for 20 days (mmol·L^−1^)	2.47 ± 0.25	2.58 ± 0.29	2.74 ± 0.36
Glycogen for 40 days (mmol·L^−1^)	3.19 ± 0.45^b^	4.25 ± 0.51^a^	3.38 ± 0.42^b^

*Diverse little letters show significant differences (P < 0.05) in different groups with Duncan's multiple range test*.

**Table 3:** Serum biochemistry parameters in juvenile GIFT injected by PBS, miR-1338-5p antagomir, or negative control for 40 days.

	**PBS**	**miR-1338-5p antagomir**	**Negative control**
**SERUM PARAMETERS (*****n*** = **12 REPLICATES PER GROUP)**			
TC for10 days (mmol·L^−1^)	3.15 ± 0.40	3.02 ± 0.21	3.22 ± 0.52
TC for 20 days (mmol·L^−1^)	3.21 ± 0.62	3.69 ± 0.41	3.59 ± 0.47
TC for 40 days (mmol·L^−1^)	3.85 ± 0.69^b^	4.51 ± 0.62^a^	3.94 ± 0.68^b^
TG for 10 days (mmol·L^−1^)	2.47 ± 0.16	2.59 ± 0.62	2.71 ± 0.53
TG for 20 days (mmol·L^−1^)	2.69 ± 0.38	2.83 ± 0.54	2.79 ± 0.69
TG for 40 days (mmol·L^−1^)	3.04 ± 0.64^c^	4.17 ± 0.49^a^	3.56 ± 0.52^b^
GLU for10 days (mmol·L^−1^)	2.31 ± 0.38	2.44 ± 0.19	2.26 ± 0.37
GLU for 20 days (mmol·L^−1^)	2.93 ± 0.49^b^	3.54 ± 0.37^a^	3.12 ± 0.61^ab^
GLU for 40 days (mmol·L^−1^)	3.15 ± 0.38^b^	4.85 ± 0.31^a^	3.52 ± 0.49^b^
Gh for 10 days (ng.mL^−1^)	1.78 ± 0.14	1.69 ± 0.17	1.83 ± 0.21
Gh for 20 days (ng.mL^−1^)	2.28 ± 0.38^a^	1.59 ± 0.32^b^	2.17 ± 0.41^a^
Gh for 40 days (ng.mL^−1^)	2.56 ± 0.19^a^	1.82 ± 0.32^b^	2.47 ± 0.33^a^
Insulin for 10 days (mIU.L^−1^)	2.23 ± 0.17	2.15 ± 0.23	2.31 ± 0.19
Insulin for 20 days (mIU.L^−1^)	2.56 ± 0.38^a^	2.19 ± 0.29^b^	2.47 ± 0.32^ab^
Insulin for 40 days (mIU.L^−1^)	2.63 ± 0.42^a^	2.04 ± 0.34^b^	2.81 ± 0.51^a^

*Diverse little letters show significant differences (P < 0.05) in different groups with Duncan's multiple range test*.

## Discussion

*ghitm* is a growth hormone-induced gene that was first found in the adipose tissue of a dwarf mouse line, because of its expression by *gh* induction (Li et al., 2001). Ghitm could mediate the neuroprotective effects of the Gh–Igf-I axis on the central nervous system of animals (Harvey and Baudet, 2011). Ghitm was distributed in T lymphocytes of poultry and that *ghitm* mRNA expression was regulated by chemokines (Nagel et al., 2004). In a study of the development of the chicken immune system, Ghitm regulated growth and was one of the specific genes of B lymphocytes (Koskela et al., 2003). Ghitm has also been found to play an important role in invertebrates. The prothoracic gland-derived receptor of a Ghitm ortholog was found to be involved in molting and metamorphosis of silkworm *Bombyx mori*, and *ghitm* mRNA expression in *D. melanogaster* was regulated by its aging state (Yoshida et al., 2006). In sea cucumber, lipopolysaccharide stimulation induced the expression of *ghitm* mRNA in the lenticular cells, activated the immune response, and enhanced its anti-infective ability (Gao et al., 2014). Ghitm has complex biological functions, and plays key roles in animal growth and regulation of immune systems.

The completion of the genome sequencing of Nile tilapia has provided favorable conditions for studying the functions of specific genes and regulatory networks (Soler et al., 2010). In our previous study, we found that metabolism and the immune system were important response pathways of GIFT under heat stress by an integrated analysis of miRNAs and mRNAs (Qiang et al., 2017a). In particular, our sequencing results showed that the absolute quantitative value of miR-1338-5p had a significant inhibitory effect in the heat-stressed group. miR-1338-5p-mediated biological pathways included mainly cell growth, developmental, and oxidative systems by the Kyoto Encyclopedia of Genes and Genomes analysis (Qiang et al., 2017a). We used bioinformatics software and the complete Nile tilapia genome sequence to predict the potential target gene of miR-1338-5p, and found that the *ghitm* 3′-UTR (351–357 bp) from GIFT completely matched a 7-nt “seed sequence” that mapped to the 5′ end of the miR-1338-5p sequence (2–8 site). Based on the results of the transcriptome sequencing, we speculated that miR-1338-5p may mediate *ghitm* regulation. Therefore, in this study, we conducted a series of experiments to verify the binding site and functional role of the miR-1338-5p-*ghitm* pair.

The luciferase reporter assay is an important miRNA site identification method that is often used to verify whether miRNAs bind directly to the 3′-UTRs of their potential target genes (Enright et al., 2003). To confirm whether *ghitm* was regulated by miR-1338-5p, we synthesized the 3′-UTR fragment containing the seed sequence into a reporter vector to obtain the *ghitm*-WT reporter vector, and constructed a vector containing the mutant sequence (*ghitm*-Mut). The constructed recombinant plasmids and miR-1338-5p were transiently transfected into HEK 293T cells. The luciferase activity of the *ghitm*-WT 3′-UTR+miRNA-1338-5p reporter gene vector was significantly lower than the luciferase activity of the control and *ghitm*-Mut groups. Also, northern blot, qRT-PCR, and injection of the miR-1338-5pantagomir showed that the expression levels of miR-1338-5p and *ghitm* were negatively correlated, which is consistent with the relationship between miRNA and target gene pairs in animals (Qiang et al., 2017a). The above results demonstrate that *ghitm* can act as a target gene for miR-1338-5p. Pituitary and liver are important organs involved in regulating the growth and metabolism of fish (Qiang et al., 2012). High miR-1338-5p levels may be involved in the regulation of hormones and energy metabolism in fish, and may intervene in growth and development.

miRNAs are broad regulatory factors that usually regulate a number of genes. They specifically interact with the 3′-UTRs of their target genes, and affect mRNA translation (Bartel, 2004; Chen et al., 2010). miR-1, miR-133a, and miR-206 are development-related miRNAs that have been widely studied. miR-1 regulates the expression of the muscle growth inhibitory factor histone deacetylase 4 gene, thereby promoting muscle growth (Chen et al., 2005). Paired box 7 (*Pax7*) was a direct target gene of miR-1 and miR-206, and their interaction affected the development of muscle cells by modulating the expression of *Pax7* (Chen et al., 2010). miR-133 can promote the proliferation of muscle cells by targeting the gene encoding serum response factor (Chen et al., 2005). miR-206 can directly regulate the expression of *igf-I* in tilapia, and inhibition of miR-206 significantly promoted the expression of *igf-I* and improved growth performance (Yan et al., 2013b). Myogenic differentiation (*myod*) is a specific gene that regulates muscle development in tilapia, miR-203b bound to the *myod* 3′-UTR. Inhibition of miR-203b increased *myod* expression, thereby stimulating the expression of development-related genes in muscle (Yan et al., 2013a). We discovered for the first time that miR-1338-5p was involved in the growth and development of GIFT by regulating the expression of *ghitm* in the pituitary and liver tissues.

Fish growth is regulated by the Gh–Ghr–Igf axis. When studying the relationship between the Gh–Ghr–Igf axis and growth of GIFT, the levels of pituitary *gh*, and hepatic *ghr1* and *igf-I* were positively correlated with growth and feed efficiency (Qiang et al., 2012). Under the same culture conditions, the growth of male Nile tilapia was 30% faster than that of female, and the expression of hepatic *ghr1* mRNA in the male fish was significantly higher than in the female, whereas *ghr1* mRNA expression in muscle was not significantly different between the sexes (Ma et al., 2009). The expression of *ghr* mRNA was significantly decreased after 2 weeks of fasting in gilthead sea bream (*Sparus aurata*), while the expression of *ghr* mRNA in muscle was unchanged. These results suggested that liver was the main target organ of fish Gh, and the amount of Ghr and level of *ghr* mRNA expression in the liver may, to some extent, reflect the growth status of fish (Alfonso et al., 2005). In this study, hepatic *ghr1* expression in GIFT was significantly decreased at 10 and 20 days after injection of miR-1338-5p antagomir. Inhibiting miR-1338-5p may enhance the binding of *ghitm* to *gh* and interfere with the regulation of *ghr1* and *igf-I* by *gh*, which may have affected the growth of GIFT in the antagomir group. However, the expression levels of pituitary *ghitm* and hepatic *ghr1* in the antagomir group at 40 day did not show the same changes at 20 day. This may be because many miRNAs have a common target gene, and inhibition of one miRNA may be compensated by other miRNAs, resulting in adaptive regulation (Gurtan and Sharp, 2013). We found that the expression levels of *gh, ghr1*, and *ghitm* were significantly increased with the prolongation of rearing time in the in PBS and NC groups, which may be related to the changes during the growth and development stage. At this stage, GIFT secrete large amounts of Gh, which activates the target cell membrane receptor or transmembrane protein to achieve its biological effects. The expression level of pituitary *gh* in the antagomir group was significantly lower than in the PBS and NC groups at 40 day, suggesting that the decrease of *ghitm* and *ghr* inhibited *gh* expression. The results of this study further suggest a synergistic and mutually restrictive relationship between *ghitm* and *ghr*, and *gh*.

In teleost fishes, the plasma Gh and Igf-I levels and their mRNA expression in liver are strongly correlated with the ratio of dietary protein to energy, dietary protein and fat source, and dietary carbohydrate levels (Enes et al., 2010; Qiang et al., 2016). Also, in the process of adipocyte differentiation, inhibition of *gh* expression can promote the expression of signal transducer and activators of transcription 5 and reduce lipolysis, thereby increasing the fat content (Sheridan, 1986; Woelfle et al., 2003). The expression of hepatic *gh* and *igf-I* in mirror carp (*C. carpio*) were inhibited by feeding a high carbohydrate diet, which promoted the expression of fat-related genes, and the crude fat content of the experimental fish was significantly higher than that of the control group (Li et al., 2015). In our previous study, we also found that high levels of serum Gh in GIFT promoted the expression of glucokinase, glucose-6-phosphatase, and glucose-6-phosphate dehydrogenase, thereby increasing glycogen utilization (Qiang et al., 2016). In this study, the high *ghitm* expression level in the miR-1338-5p antagomir group may interfere with regulation of the *gh*–*ghr*–*igf* axis in pituitary and liver. Inhibition of circulating Igf-I and binding sites of Ghr in liver can decrease serum Gh levels, thus affecting the expression of genes involved in glycogen and fat synthesis, and promoting storage of liver glycogen and fat.

Insulin is the most important regulator of blood glucose balance, and insulin mainly promotes glycogen, fat, and protein synthesis while inhibiting gluconeogenesis, thereby reducing blood glucose levels (Mommsen and Plisetskaya, 1991). In mammals, the problem of dual pathology (metabolic disorders of both glucose and lipids) caused by insulin secretion deficiency is characterized mainly by insulin resistance leading to increased blood sugar levels for glucose metabolic disorders, and to increased TG and maybe low-density lipoprotein cholesterol, and decreased high-density lipoprotein cholesterol resulting in type 2 diabetes and hypertriglyceridemia for lipid metabolic disorders(Grill and Qvigstad, 2000; Goldberg, 2001). Compared with mammals, glucose tolerance of fish is low, mainly because of low insulin secretion (Poitout et al., 2006). Differences in fish feeding habits also affect insulin secretion. Insulin secretion and glucose absorption rate of cobia (*Rachycentron canadum*) was lagging behind the GIFT, so that the absorption of glucose cannot be better use (Liu et al., 2015). In this study, inhibition of serum insulin levels in the miR-1338-5p antagomir group may interfere with fat and glucose utilization. In mammals, insulin is an important anti-lipolytic and adipogenic hormone, which may have a similar function in fish. The inhibition of the signaling pathway in the *gh*–*ghr*–*igf* axis led to decreased serum Gh levels that may affect insulin secretion, and caused over up-regulation of lipid metabolism in the fat cells resulting in obesity or promoting gluconeogenesis resulting in elevated blood glucose levels (Hwu et al., 1997; Lu et al., 2013).

## Conclusion

For the first time, this study confirmed that miR-1338-5p could be used as a new regulator of growth and metabolism in GIFT. miR-1338-5p can bind to the *ghitm* 3′-UTR and their expression levels have a negative relationship. By interfering with signal transduction in the growth axis, the growth and metabolism of GIFT were affected. Inhibition of miR-1338-5p promoted the expression of *ghitm* in pituitary and liver. *ghitm* could compete with *ghr1* in combining to *gh*, thereby interfering with the *gh*–*ghr1*–*igf-I* signaling pathway, reducing Gh secretion, causing insulin antagonism, and increasing glycogen and fat synthesis. The results of this study will help to better understand the miR-1338-5p-*ghitm* regulators involved in regulating the fish growth process. In the next study, we will investigate the possible involvement of the miR-1338-5p-*ghitm* pair in other biological signaling pathways (e.g., oxidative reactions and neural signals) to better understand the regulatory pathways and the response mechanism of miR-1338-5p-*ghitm* regulators *in vivo*.

## Author contributions

PX and JQ conceived and designed the experiment; HL conceived and implemented the database; JB and YT sampled the liver and pituitary tissues, and extracted RNA; JH carried out the functional analysis of miRNA; DC analyzed biochemical indicators in serum and liver; JQ wrote the paper with contributions from YT, JB, PX, JH, and DC. All authors read and approved the final version of the manuscript.

### Conflict of interest statement

The authors declare that the research was conducted in the absence of any commercial or financial relationships that could be construed as a potential conflict of interest.
